# Study protocol for a parallel-group randomized controlled trial of internet-delivered behavior therapy for adults with Tourette syndrome

**DOI:** 10.3389/fdgth.2025.1518666

**Published:** 2025-08-29

**Authors:** Max Sannemalm, Nathalie Lybert, Lisa Gunnarsson, Per Andrén, Martin Kraepelien, Maria Bragesjö, Robin Fondberg, Volen Z. Ivanov, David Mataix-Cols, Lorena Fernández de la Cruz, Erik Andersson, Christian Rück, Ekaterina Ivanova

**Affiliations:** ^1^ Centre for Psychiatry Research, Department of Clinical Neuroscience, Karolinska Institutet & Stockholm Health Care Services, Region Stockholm, Stockholm, Sweden; ^2^ Department of Psychiatry, University of Oxford, Oxford, United Kingdom; ^3^ Department of Clinical Sciences, Lund, Lund University, Lund, Sweden; ^4^ Division of Psychology, Department of Clinical Neuroscience, Karolinska Institutet, Stockholm, Sweden

**Keywords:** Tourette syndrome, chronic motor or vocal tic disorder, behavioral therapy, internet-delivered behavior therapy, exposure and response prevention, randomized controlled trial, health economics

## Abstract

**Introduction:**

Tourette syndrome (TS) and chronic motor or vocal tic disorder (CTD) are neurodevelopmental disorders associated with functional impairment and reduced quality of life. Behavioral therapy (BT) is an effective treatment, but lack of experienced practitioners makes it hard for patients to receive appropriate help. One approach to bridge the gap between demand and availability is to offer the treatment remotely over the internet with minimal support from a therapist.

**Methods:**

This single-blind randomized controlled superiority trial including 110 participants will compare internet-delivered BT (I-BT) primarily consisting of exposure and response prevention (ERP) to a control condition consisting of internet-delivered general psychological support. The primary aim of the trial is to evaluate whether ERP-based I-BT is superior to the control condition in reducing TS/CTD symptoms. The primary outcome measure is the Yale Global Tic Severity Scale - Total Tic Severity score administered by blinded raters at primary endpoint 11 weeks after the treatment start. Secondary endpoints occur at week 23 and 14 months after the treatment start, and the secondary outcomes include tic-related impairment, rates of responders, self-rated tic severity, symptoms of depression, quality of life and cost-effectiveness. Data on dropout rates and adverse events is also collected.

**Discussion:**

This is the first randomized controlled trial to evaluate therapist-guided ERP-based I-BT for adults with TS/CTD. The study has been approved by the Swedish Ethical Review Authority (EPM 2023-06541-01). The hypotheses were pre-registered before the start of the data collection. Results from all analyses will be reported according to the Consolidated Standards of Reporting Trials statement for non-pharmacological trials (CONSORT) and Consolidated Health Economic Evaluation Reporting Standards (CHEERS). The participants in the control condition will have the opportunity to receive I-BT after the data from the first follow-up is collected. The study will be published in open access and the results will be shared with service user organizations. At the moment of submission, the study has recruited 87 out of 110 planned participants and the recruitment is expected to be completed in February 2025.

**Trial registration:**

Open Science Framework: https://osf.io/cq97b/ (uploaded 31/01/2024); Clinicaltrials.gov: NCT06271083 (submitted 14/02/2024).

## 1 Introduction

Tourette syndrome (TS) and chronic motor or vocal tic disorder (CTD) are neurodevelopmental conditions that originate and typically peak in childhood (1), but about 20% of patients seem to present with the symptoms beyond their teenage years (2, 3). TS is characterized by both motor and vocal tics for at least one year, while CTD involves either motor or vocal tics also lasting at least one year (1). Both conditions are associated with reduced quality of life, lower educational attainment, risk of other psychiatric and somatic conditions, and increased mortality rates (3–10).

The most common pharmacological treatment for TS/CTD is antipsychotic medication, which is associated with substantial side effects (11). Behavior therapy (BT) is a non-pharmacological treatment option aimed at teaching individuals to better manage their tics (12). BT is an effective treatment (13–16), but is not always available due to a lack of experienced practitioners.

One approach to increase treatment availability is to offer BT online instead of in-person (17). Internet-delivered BT (I-BT) includes the same treatment content but is delivered through a secure online platform. Patients work with self-help materials and assignments and communicate with a therapist via the platform. Research shows that self-help-based I-BT can be provided to adults with TS/CTD (18). Therapist-supported I-BT using exposure and response prevention (ERP) techniques has been successfully provided to children and adolescents in two large randomized controlled trials (19, 20) with one trial showing significant reductions in tic severity compared to an active control group (19), and the second trial showing significant reductions in both groups with no between-group effect (20). Our research group recently conducted a feasibility trial of internet-delivered ERP for adults (*n* = 31) with TS/CTD (21). The treatment was deemed both feasible and safe. Additionally, preliminary efficacy results showed a significant within-group tic severity reduction, with an effect size of Cohen's d = 0.49.

The primary aim of the study is to investigate whether guided internet-delivered ERP-based BT is superior to an active control condition consisting of guided internet-delivered general psychological support in reducing TS/CTD symptoms. We hypothesize that, compared to the control group, participants in the treatment group will show greater reduction in tic severity, as measured with Yale Global Tic Severity Scale - Total Tic Severity subscale (YGTSS-TTS) (22) at the primary endpoint (post treatment or week 11 after treatment start).

The secondary aim at the primary endpoint (week 11 after the start of the treatment) is to compare the two arms regarding:

(1) Tic-related impairment
(2) Rates of responders
(3) Self-rated tic severity
(4) Symptoms of depression
(5) Quality of life
(6) Cost-effectiveness

The third aim is to evaluate long-term maintenance of gains. We hypothesize that the intervention condition will be superior to the control condition at week 23 after the treatment start (better response, less severe symptoms, higher quality of life) regarding all outcome measures and that the results will be maintained in the intervention group at the 14-month follow-up. The fourth aim is to evaluate the cost-effectiveness of the intervention from the healthcare organization payer perspective, the healthcare system perspective, and the societal perspective.

## 2 Methods and analysis

### 2.1 Trial design

**Figure 1:**
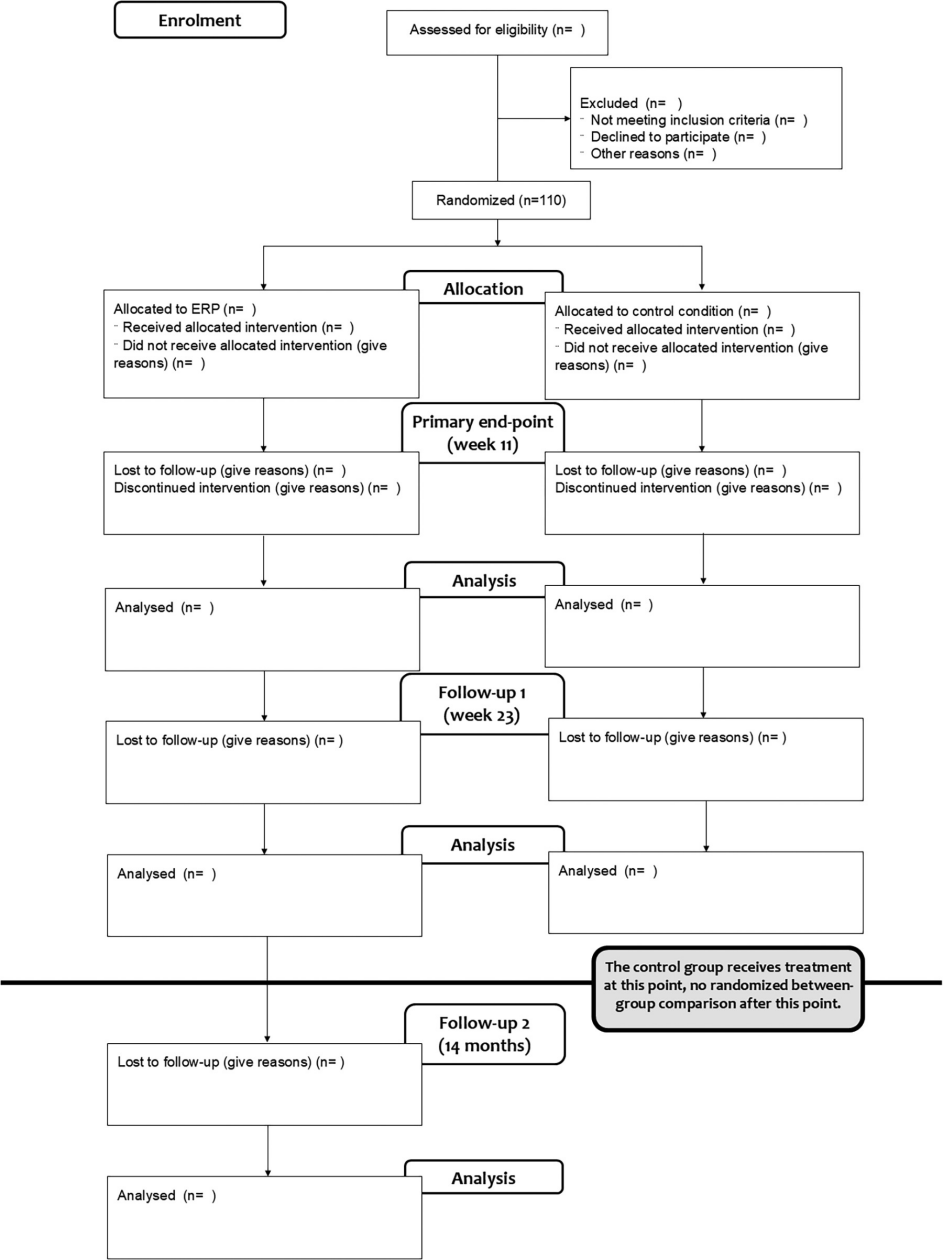
CONSORT flow chart over the trial recruitment and treatment process.

A parallel group randomized (1:1) controlled superiority trial (RCT) will be carried out at Karolinska Institutet in Stockholm, Sweden. The study will be conducted in Swedish and will include a total of 110 adult participants with TS/CTD. The study will be conducted according to the Declaration of Helsinki and relevant parts of ICH E6 Good Clinical Practice (GCP). The Consolidated Standards of Reporting Trials (CONSORT) flow chart for the trial is shown in Figure 1. Both the outcome measures and the treatment will be administered via a secure online treatment platform (BASS4) requiring two-step authentication, run by the Karolinska Institutet eHealth Core Facility. Prior to the start of recruitment, the trial was approved by the Swedish Ethical Review Authority (EPM 2023-06541-01) and pre-registered at Open Science Framework, osf.io (https://osf.io/cq97b/). The trial was registered at ClinicalTrials.gov (NCT06271083) at the very start of recruitment. Potential protocol modifications will be described in detail on osf.io, ClinicalTrials.gov, and, if required, amendments will be submitted to the Swedish Ethical Review Authority.

### 2.2 Participants

Eligibility criteria for the participants are listed in Table 1. The participants will be recruited nationally by means of advertisement in social and regular media (online and printed copies) and by spreading information via psychiatric and neurological clinics and service user organizations across Sweden. The advertisements will guide the prospective participants to the study website (ticsstudien.se) where more detailed information about the study will be provided and the visitor will be encouraged to proceed to the treatment platform for registration. During the registration procedure, the prospective participants will be provided necessary practical information about the project. Thereafter, if they are still interested in participating, they will provide digital consent for study participation, identify themselves with two-step authentication by receiving a text message to their phone number and e-mail address, create an account at the treatment platform, and fill out a preliminary screening form focused on the nature and severity of their tics, the eligibility criteria, and sociodemographic information. Potentially eligible participants will undergo a psychiatric assessment via video-conferencing software or telephone to check the eligibility criteria, collect clinician-administered measurements, and assess psychiatric comorbidities. Eligible participants will be included in the trial, randomized, and enrolled within two weeks after the assessment of pre-treatment tic severity with the YGTSS (if more than two weeks pass from the date of pre-treatment YGTSS to the start of the treatment, the pre-treatment YGTSS will be re-administered).

**Table 1:** Overview of eligibility criteria.

Type of eligibility criteria	Eligibility criteria
Inclusion criteria	≥18 years of age.
	Primary diagnosis of TS/CTD, according to DSM-5 criteria.
	Provided digital informed consent.
	A Total Tic Severity Score (TTS) of >15, or >10 for individuals with motor or vocal tics only, in the past week, as measured by the Yale Global Tic Severity Scale (YGTSS).
	Willing and able to follow the study procedures and participate in the 10-week treatment program.
	Fluent in Swedish.
	Regular access to a computer connected to the internet, sufficient technical skills to use the treatment platform, as well as a mobile phone to receive text messages.
Exclusion criteria	Ongoing or planned psychological treatment for TS/CTD.^a^
	Previous BT for TS/CTD of a minimum of 8 sessions with a qualified therapist within 12 months prior to assessment.^a^
	Adjustment of medication for TS/CTD within the last two months prior to assessment.^a^
	Severe psychiatric comorbidities such as organic brain disorders, bipolar disorder, ongoing psychosis, anorexia nervosa or substance use disorders that may interfere with the treatment for TS/CTD.^a^
	Acute psychiatric problems such as severe depression or suicidal risk needing immediate psychiatric care.
	Severe tics causing immediate risk to the participants themselves or to others and requiring urgent medical attention.

DSM-5, diagnostic and statistical manual of mental disorders, fifth edition; TS/CTD, Tourette syndrome/ chronic tic disorder; YGTSS, Yale Global Tic Severity Scale; YGTSS-TTS, Yale Global Tic Severity Scale—Total Tic Severity subscale.

^a^ These criteria are selected for exclusion as these factors can interfere with the treatment.

### 2.3 Interventions

#### 2.3.1 Intervention condition: internet-delivered exposure with response prevention

The active intervention will contain eight chapters with homework assignments after each chapter, as well as working sheets for the participants to register and monitor their activity during the treatment. The participants will work with their treatment for 10 weeks, communicating with their therapists via asynchronous written messages in the platform. On the first day of the treatment, the participants in both intervention and control conditions will be greeted by their therapist via a message sent on the treatment platform. The therapists will introduce themselves, inform the participants about the outline of the treatment, and encourage them to start working with the treatment content immediately. The first two chapters of the treatment will be available to the participants from the start. The participants will be encouraged to work with one treatment chapter per week and the following chapter will be opened by the therapist when the previous week's homework is reviewed. The therapist's role will be to encourage the participants’ activity in the treatment and follow up on inactivity, problem solve and follow up on potential safety issues when necessary.

During the treatment, participants will receive information about tics and ERP, the central component of the intervention (see Table 2 for an overview of the ERP condition). Continuously throughout the treatment, participants will work with exposing themselves to situations that trigger their premonitory urges and practice suppressing their tics. They will learn different strategies to provoke their premonitory urges to make suppression of their tics more challenging and gradually increase the time they can suppress the tics. A central tool in the treatment will be the Ticstimer, a worksheet where participants will continuously record their ERP practice and the time they manage to suppress tics. The participants will be encouraged to work with the Ticstimer daily. Additionally, the treatment will include an introduction to habit reversal training (HRT) which is another BT-strategy, to give the participants and therapists a broader range of tools and make the treatment more suitable for regular psychiatric settings where clinicians appreciate being able to offer a range of evidence-based techniques. The treatment will include one scheduled phone call around the time the participant received the rationale for the ERP and is about to start their practice, to maximize the chance for the first practice to align with the rationale.

**Table 2:** Overview of the active condition.

Chapter 1—Introduction. Provides practical information about the treatment (length, homework assignments, therapist support), and introduces the psychological model for maintenance of tics.
Chapter 2—Register the urges. Introduces the concept of premonitory urges and encourages the participants to start registering the urges as a part of their training.
Chapter 3—Exposure with response prevention. Introduces the participants to exposure and response prevention and encourages them to start using the Ticstimer.
Chapter 4—Proactive exposure Part 1. Encourages the participants to expose themselves to more challenging situations to provoke tics and try to practice suppressing tics.
Chapter 5—Proactive exposure Part 2. The participants are encouraged to get more creative to increase the difficulty level of the exercise, for example by inducing tics and then trying to interrupt them. Habit reversal strategies are introduced.
Chapter 6—Proactive exposure Part 3. The level of difficulty increases even further, as the participants are encouraged to combine different exercises and minimize avoidance of triggering situations in their everyday life.
Chapter 7—Summary of the treatment. Summarizes the core components of the treatment.
Chapter 8—Planning forward. Planning for continued work and relapse prevention.

#### 2.3.2 Control condition: internet-delivered general psychological support

Participants who are randomized to the control group will receive access to brief psychoeducational content about TS/CTD in the platform in combination with general psychological support from a therapist. The materials will be divided in two chapters. Chapter 1 will cover the topics of what tics are, and the different ways stress can influence the symptoms. Chapter 2 will focus on supporting stress management and healthy lifestyle habits related to food, sleep, and physical activity. No active BT components (i.e., ERP, HRT, applied relaxation) are provided to the participants in the control group. The therapists will welcome the participants to the treatment, encourage them to engage in the treatment content and to reach out to the therapist if they have any questions. Thereafter, the therapists will only reply to the participants’ messages without taking any contact initiative themselves (unless there is an indication of severe deterioration or severe depressive symptoms or suicidal ideation requiring medical attention). The aim of the control condition is to control for basic therapist attention, relevant disorder-specific content, and the passage of time. The participants in the control group who wish to have additional help will have an opportunity to receive I-BT after the data from the first long-term follow-up (week 23 after the start of the treatment) are collected.

#### 2.3.3 Therapists

The therapists will answer participants’ messages within 24 h on weekdays. The therapists will be licensed psychologists and psychologists or psychology students under supervision and with basic training in cognitive behavior therapy. All therapists will be treating participants in both conditions. The therapists will receive a half-day training in the program followed by supervision sessions every two weeks throughout the duration of the trial with an experienced therapist.

### 2.4 Outcomes

If not specified otherwise, the outcome measures will be collected at pre-treatment, at week 11 counting from the start of the treatment (post-treatment, primary endpoint), week 23 (3 months after the end of the treatment), and 14 months after the treatment start (12 months after the end of the treatment) (Table 3).

**Table 3:** Overview of the recruitment, treatment, and assessment points and measures.

	Enrolment	Allocation	Pre-treatment	Week 1–3	Week 4–7	Week 8–10	Week 11 (post-treatment, primary endpoint)	Week 23	14-month follow-up
Enrolment									
Eligibility screen	X								
Informed consent	X								
Allocation		X							
Treatment									
ERP				X	X	X			
Control condition				X	X	X			
Clinician-administered measures									
DSM-5 criteria for TS/CTD	X								
SCID-5	X								
MINI	X								
YGTSS	X						X	X	X
CGI-S	X						X	X	X
CGI-I							X	X	X
iiPAS					X		X		
Self-rated measures									
ATQ1	X		X	X	X	X	X	X	X
RAADS-14			X						
ASRS			X						
PUTS			X						
MADRS-S^b^	X		X	X	X		X	X	X
AUDIT	X								
DUDIT	X								
WSAS^a^			X	X	X	X	X	X	X
GTS-QoL			X				X	X	X
AQoL-6D			X				X	X	X
TIC-P			X				X	X	X
Negative effects							X	X	
Other adverse events				X	X		X	X	
CEQ					X				
WAI-SR					X				
Time spent on the programme^a^				X	X	X	X	X	X

AQoL-6D, assessment of quality of life—6 dimensions; ASRS, adult ADHD self-report scale; ATQ, adult tic questionnaire; AUDIT, alcohol use disorders identification test; CEQ, credibility and expectancy questionnaire; CGI-I, clinical general impression—improvement; CGI-S, clinical general impression—severity; DUDIT, drug use disorders identification test; ERP, exposure with response prevention; GTS-QoL, gilles de la Tourette syndrome—quality of life scale; iiPAS, internet interventions patient adherence scale; MADRS-S, montgomery—asberg depression rating scale—self-rated; MINI, the mini international neuropsychiatric interview; PUTS, premonitory urge for tics scale; RAADS-14, ritvo autism and asperger diagnostic scale; SCID-5, OCD and related disorders section of the Structured Clinical Interview for DSM-5; TIC-P, treatment inventory of costs in patients with psychiatric disorders; TS/CTD, Tourette syndrome/chronic tic disorder; WAI-SR, working alliance inventory—self-rated; WSAS, work and social adjustment scale.

^a^ Administered weekly during the 10-week treatment.

^b^ Administered at week 3 and 7.

The primary outcome measure will be the YGTSS-TTS (22), administered by a clinician blinded to the randomization condition. The raters will be psychiatrists or psychiatry residents, clinical psychologists, psychologists under training or psychology students receiving supervision. The raters will receive training in administering the YGTSS and participate in at least two test ratings with subsequent supervision before they are allowed to conduct assessments in the trial. Co-ratings will be arranged every 6 months for the active raters during the data collection period. Data on inter-rater reliability will be reported.

Secondary clinician-rated outcome measures will be the YGTSS-Impairment, as well as the Clinical Global Impression—Severity and Improvement (CGI-S and CGI-I, the latter not administered at pre-treatment) (23). The clinicians will receive training in administering the CGI that will be repeated every 6 months. Participants that are rated as “much improved” or “very much improved” on the CGI-I will be classified as treatment responders (24). If the participant keeps the responder status at subsequent follow-ups, they will be classified as long-term responders at that follow-up.

Self-rated tic-specific outcome measures will be the Adult Tic Questionnaire (ATQ) (also administered weekly) (25), the Gilles de la Tourette Syndrome—Quality of Life Scale (GTS-QoL) (26), and the Work and Social Adjustment Scale (WSAS) (27) adapted for TS/CTD by the research team. General quality of life will be measured using the Assessment of Quality of Life—6 Dimensions (AQoL-6D) (28), and these scores will be used in the health-utility analysis (see below). Symptoms of depression will be assessed using Montgomery-Asberg Depression Rating Scale—Self-report (MADRS-S) (29) (also administered at week 3 and 7). The self-reported Treatment Inventory of Costs in Patients with psychiatric disorders (TIC-P) will be administered to measure health-related costs (30).

The participants’ adherence to the treatment will be measured by their therapist using the Internet Intervention Patient Adherence Scale (iiPAS) (31) at week 6 and 11. In the ERP condition, once the Ticstimer is introduced, the participants will be asked to assess weekly how much time they have spent practicing. The participants will rate the perceived credibility of the treatments using the Credibility and Expectancy Questionnaire (CEQ) (32) at week 5. The participants will also fill out the Working Alliance Inventory—Self-Report (WAI-SR) (33) at week 5. Data on the number of sent and received messages, phone calls, and completed treatment chapters will be collected, as well as data on time spent by the therapists on supporting each participant.

The following instruments will only be administered at pre-treatment: The Mini International Neuropsychiatric Interview (M.I.N.I.) (34) and the OCD and related disorders section of the Structured Clinical Interview for DSM-5 (SCID) (35) to assess psychiatric comorbidities (clinician-administered); questions on tic symptoms following DSM-5 criteria for TS/CTD to confirm the diagnosis (clinician-administered), as well as the following self-rated measures: Premonitory Urge for Tics Scale (PUTS) (36), Alcohol Use Disorders Identification Test (AUDIT) (37), Drug Use Disorders Identification Test (DUDIT) (38), the Ritvo Autism and Asperger Diagnostic Scale (RAADS-14) (39), and the Adult ADHD Self-report Scale (ASRS) (40). Data on medication and previous psychological treatments will be collected at the baseline clinical assessment.

Negative effects will be assessed by administering a 16-item questionnaire on negative events developed by the research group at post-treatment and at week 23. Adverse events connected to worsened symptoms, suicidality, and impatient care will be measured using an additional questionnaire developed by the research group focusing specifically on whether those events have occurred, with possibility for elaboration, also administered at week 6. Spontaneously reported adverse events will be registered. Patient safety will be monitored during the recruitment procedures, by administering the MADRS and an adverse events questionnaire twice during the treatment, as well as by clinician monitoring in their contact with the participants. In case of significant deterioration, severe depressive symptoms or signs of suicidality, or in case of progression of other psychiatric disorder needing medical attention, the participant will be contacted for an additional assessment and an experienced clinician will decide on recommended future care (e. g., discontinuation of the treatment, referral to inpatient or other outpatient care).

To promote completion of follow up questionnaires, the participants will receive automatic reminders via text messages in the study platform. In case of longer measurement inactivity, study personnel will be contacting the participants via text messages or by phone.

### 2.5 Sample size

The within-group effect size in the feasibility trial was Cohen's d = 0.49 (21). As tic severity is known to fluctuate over time, we expect a certain amount of improvement in the control condition. However, we do not expect it to be as large as in the two previous ERP-based I-BT studies in children (19, 20), because the control group in those studies was much more potent. Furthermore, the participants in our previous adult pilot trial improved as much as the participants in a previous adult trial (18), but their pre-treatment tic severity was unexpectedly low, making the overall pre-to-post-treatment effects small. In the current trial, we expect the participants to have a more representative pre-treatment tic severity and their improvement to the primary endpoint is expected to be larger. In sum, we assume the between-group effect-size measured with Cohen's d to be around 0.5. With *n* = 50 participants in each group we will achieve 80% power to detect the effect of between-group Cohen's d = 0.5 (one-tailed test). A total sample size of *n* = 110 will allow for 10% drop-out rate.

### 2.6 Randomization and blinding

The participants will be starting treatment in cohorts. Every time a cohort is ready to start, the online service random.org will be used to create a randomized list of the participants included into the cohort. The list will be emailed to a person not associated with the research group with the instruction to choose the following: (1) where an imaginary line dividing the cohort into two approximately equal groups should be drawn, and (2) whether the group above the line should be marked with “I” (meaning intervention) or “K” (meaning control). The outcome assessors will be blinded to the allocation of the participants, and they will be asked to guess the allocation after each assessment. The frequency of correct guesses will be reported as a measure of blinding integrity.

### 2.7 Statistical methods

The significance level for all statistical tests will be *α* = 0.05. For all scale level measures, Cohen's d will be used as a measure of effect size. Odds ratios will be reported for categorical variables. Confidence intervals (95%) will be reported for all estimated measures.

The intention-to-treat approach to the analysis population will be applied in all cases unless specified otherwise. In case more than 10% of the data is missing, maximum likelihood imputations will be performed based on demographical data and the scores from the pretreatment assessment. The between-group differences at week 11 (primary endpoint) for the primary (YGTSS-TTS) and secondary outcome measures, an analysis of covariance (ANCOVA) with pre-score of the measure as covariate will be calculated. For ATQ and WSAS that are collected weekly, mixed effect models will be applied with random intercept and random slope. Chi squared tests will be calculated to explore the between-group differences in proportions of responders and long-term responders at all endpoints.

### 2.8 Health-economic evaluation

We will conduct a within-trial health economic evaluation (41) whereby outcome and cost data will be compared at week 11. It will encompass two analyses: (1) a cost-effectiveness analysis using the responder status as outcome; (2) a cost-utility analysis using the outcome quality adjusted life years (QALYs) measured using the AQOL-6D (30).

Health-related quality of life (HRQoL) will be collected using the AQOL-6D (28, 42), a multi-attribute utility instrument for use in economic evaluations. Data on resource use will be collected using an adapted version of the self-reported Treatment Inventory of Costs in Psychiatric Patients (TIC-P) questionnaire (36). Costs will be measured from three perspectives: a healthcare organization payer perspective (including intervention costs only), a healthcare system perspective (additionally including costs related to the use of medical resources, and medication), and a societal perspective (additionally including social care costs and productivity losses for the individual). Costs from each perspective will be analysed in relation to clinical efficacy (responder status; cost-effectiveness analysis) and QALYs (cost-utility analysis). Results will be presented as incremental cost-effectiveness ratios (ICER), as the ratio between the difference in costs and the difference in health outcomes ICERs will be put against values of willingness to pay for a QALY to determine cost-effectiveness.

Standard health economic evaluation techniques will be used to explore uncertainty around the cost and effect data, which will be represented on cost-effectiveness planes. The probability of cost-effectiveness according to different willingness to pay thresholds will be represented on cost effectiveness acceptability curves (43).

## 3 Discussion

This will be the first superiority trial to compare a therapist-guided digital I-BT programme, primarily consisting of ERP, for adults with TS/CTD to an active control group. This trial aims to provide a population that rarely has access to effective treatments with an evidence-based intervention that can be widely disseminated.

## 4 Ethics and dissemination

This study has been approved by the Swedish Ethical Review Authority (EPM 2023-06541-01). The benefits of the project outweigh its potential harms. The treatment is built on the same evidence-based principles shown to be safe in previous trials and in our own pilot trial (21). The participants in the control condition will have a chance to receive the treatment after the data from the first follow-up are collected. The participants’ symptoms will be closely monitored by the study personnel. At signs of deterioration or worsening of symptoms of depression or suicidal ideation, the participant will be assessed by a licensed clinical psychologist or psychiatrist. If necessary, the trial participation will be ended for these participants, and they will be referred to adequate medical services. All participant data will be collected and stored at either the encrypted treatment platform BASS4, run by the Karolinska Institutet eHealth Core Facility and requiring two-step authentication, or at the encrypted servers at Karolinska Institutet, set up specifically for storing sensitive research data and requiring a VPN-connection. There will be no external data monitoring committee in this trial. All data will be monitored in-house by study personnel. Participants always have the right to discontinue treatment at any time, without needing to provide an explanation. The research team is not allowed to modify the intervention, but the participants are not prohibited to do so, meaning their treatment will not be automatically discontinued if they engage in another treatment.

The publications within the current project will be published in Open Access journals. The statistical code for the analyses will be uploaded at osf.io (https://osf.io/cq97b/) and a detailed description of the dataset will be saved in Swedish National Database (SND).

## 5 Current trial status

The first participant was included in the study on the 8th of February 2024 and recruitment is expected to end in January 2025. At the time for the submission, 87 participants have been included in the study. The last follow up will preliminarily take place in July 2026. Data analysis will begin after all primary endpoint data have been collected in July 2025.
