# Supplementary material for: Study protocol for a parallel-group randomized controlled trial of internet-delivered behavior therapy for adults with Tourette syndrome
Source: Front Digit Health. 2025 Aug 29;7:1518666. doi: 10.3389/fdgth.2025.1518666 (PMC12426249; doi:10.3389/fdgth.2025.1518666)
Supplement: Supplementary file 1 [file Datasheet1.docx]

Supplementary Material

Study Protocol for a Parallel-Group Randomized Controlled Trial of Internet-Delivered Behavior Therapy for Adults with Tourette Syndrome

World Health Organization Trial Registration Data Set

| **Data category** | **Information** |
| --- | --- |
| Primary registry and trial identifying number | Open Science Framework: https://osf.io/cq97b/ |
| Date of registration in primary registry | 31 January 2024 |
| Secondary identifying numbers | Swedish Ethical Review Authority: EPM 2023-06541-01  Clinicaltrials.gov: NCT06271083 |
| Source(s) of monetary or material support | N/A |
| Primary sponsor | Karolinska Institutet, Sweden |
| Secondary sponsor(s) | N/A |
| Contact for public queries | PhD student Max Sannemalm, Karolinska Institutet, Sweden, +46736821988 [max.sannemalm@ki.se](mailto:max.sannemalm@ki.se) |
| Contact for scientific queries | PhD student Max Sannemalm, Karolinska Institutet, Sweden, +46736821988 [max.sannemalm@ki.se](mailto:max.sannemalm@ki.se) |
| Public title | *Study Protocol for a Parallel-Group Randomized Controlled Trial of Internet-Delivered Behavior Therapy for Adults with Tourette Syndrome* |
| Scientific title | Study Protocol for a Parallel-Group Randomized Controlled Trial of Internet-Delivered Behavior Therapy for Adults with Tourette Syndrome |
| Countries of recruitment | Sweden |
| Health condition(s) or problem(s) studied | Tourette syndrome and chronic tic disorder |
| Intervention(s) | Active comparator: Internet-based behaviour therapy with emphasis on exposure and response prevention for 10 weeks Control comparator: Internet-based general psychological support including brief psychoeducational content |
| Key inclusion and exclusion criteria | Ages eligible for study: ≥18 years Sexes eligible for study: both Accepts healthy volunteers: no Inclusion criteria:   1. ≥ 18 years of age. 2. Primary diagnosis of TS/CTD, according to DSM-5 criteria. 3. Provided digital informed consent. 4. Have a Total Tic Severity Score (TTS) of >15, or >10 for individuals with motor or vocal tics only, in the past week, as measured by the Yale Global Tic Severity Scale (YGTSS). 5. Being willing and able to follow the study procedures and participate in the 10-week treatment program. 6. Being fluent in Swedish. 7. Have regular access to a computer connected to the Internet, sufficient technical skills to use the treatment platform, as well as a mobile phone to receive text messages.   Exclusion criteria:   1. Ongoing or planned psychological treatment for TS/CTD. 2. Previous BT for tics of a minimum of 8 sessions with a qualified therapist within 12 months prior to assessment. 3. Adjustment of medication for tics within the last two months prior to assessment. 4. Severe psychiatric comorbidities such as organic brain disorders, bipolar disorder, ongoing psychosis, anorexia nervosa or substance use disorders that can interfere with the treatment for TS/CTD. 5. Acute psychiatric problems such as severe depression or suicidal risk needing immediate psychiatric care. 6. Severe tics causing immediate risk to the participants themselves or to others and requiring urgent medical attention. |
| Study type | Interventional Allocation: randomized Intervention model: parallel assignment Masking: single blind (outcomes assessor) Primary purpose: treatment Phase: NA |
| Date of first enrolment | 8 February 2024 |
| Target sample size | 110 |
| Recruitment status | Recruiting |
| Primary outcome(s) | Change from baseline to 11 weeks after treatment start on the Yale Global Tic Severity Scale (YGTSS), clinician-administered |
| Key secondary outcomes | - Tics-related impairment - Rates of responders/long-term responders - Self-rated tic severity - Symptoms of depression - Quality of life - Cost-effectiveness |
